# Inducible expression of human *C9ORF72* 36× G_4_C_2_ hexanucleotide repeats is sufficient to cause RAN translation and rapid muscular atrophy in mice

**DOI:** 10.1242/dmm.044842

**Published:** 2021-02-16

**Authors:** Fréderike W. Riemslagh, Esmay C. van der Toorn, Rob F. M. Verhagen, Alex Maas, Laurens W. J. Bosman, Renate K. Hukema, Rob Willemsen

**Affiliations:** 1 Department of Clinical Genetics, Erasmus University Medical Center Rotterdam, 3015 GD Rotterdam, The Netherlands; 2 Department of Cell Biology, Erasmus University Medical Center Rotterdam, 3015 GD Rotterdam, The Netherlands; 3 Department of Neuroscience, Erasmus University Medical Center Rotterdam, 3015 GD Rotterdam, The Netherlands

**Keywords:** C9ORF72, ALS, FTD, Mouse, Inducible, DPRs

## Abstract

The hexanucleotide G_4_C_2_ repeat expansion in the first intron of the *C9ORF72* gene accounts for the majority of frontotemporal dementia (FTD) and amyotrophic lateral sclerosis (ALS) cases. Numerous studies have indicated the toxicity of dipeptide repeats (DPRs), which are produced via repeat-associated non-AUG (RAN) translation from the repeat expansion, and accumulate in the brain of C9FTD/ALS patients. Mouse models expressing the human *C9ORF72* repeat and/or DPRs show variable pathological, functional and behavioral characteristics of FTD and ALS. Here, we report a new Tet-on inducible mouse model that expresses 36× pure G_4_C_2_ repeats with 100-bp upstream and downstream human flanking regions. Brain-specific expression causes the formation of sporadic sense DPRs aggregates upon 6 months of dox induction, but no apparent neurodegeneration. Expression in the rest of the body evokes abundant sense DPRs in multiple organs, leading to weight loss, neuromuscular junction disruption, myopathy and a locomotor phenotype within the time frame of 4 weeks. We did not observe any RNA foci or pTDP-43 pathology. Accumulation of DPRs and the myopathy phenotype could be prevented when 36× G_4_C_2_ repeat expression was stopped after 1 week. After 2 weeks of expression, the phenotype could not be reversed, even though DPR levels were reduced. In conclusion, expression of 36× pure G_4_C_2_ repeats including 100-bp human flanking regions is sufficient for RAN translation of sense DPRs, and evokes a functional locomotor phenotype. Our inducible mouse model suggests that early diagnosis and treatment are important for C9FTD/ALS patients.

This article has an associated First Person interview with the first author of the paper.

## Abstract (precis)

**Summary:** Only 36 C9ORF72 repeats are sufficient for RAN translation in a new mouse model for amyotrophic lateral sclerosis and frontotemporal dementia. Reducing toxic dipeptides can prevent but not reverse the phenotype.

## INTRODUCTION

Frontotemporal dementia (FTD) is a neurological disease characterized by neuronal loss in the frontal and temporal lobes, leading to behavioral and personality changes, and language deficits (Hernandez et al., 2018; Woollacott and Rohrer, 2016). The prevalence of FTD is ∼15-20 cases per 100,000 people, and the age of onset is usually between 45 to 65 years (Woollacott and Rohrer, 2016). FTD is part of a disease spectrum that also comprises amyotrophic lateral sclerosis (ALS) (Couratier et al., 2017; Strong et al., 2017). ALS is a rapid progressive motor neuron disorder that affects the upper motor neurons in the motor cortex and the lower motor neurons in the anterior horn of the spinal cord (Grad et al., 2017; Oskarsson et al., 2018). ALS patients develop muscle weakness, spasticity, atrophy and eventually paralysis (Grad et al., 2017; Oskarsson et al., 2018). The prevalence of ALS is ∼5 in 100,000 people, and the age of onset is between 50 and 60 years of age (Grad et al., 2017; Oskarsson et al., 2018). The hexanucleotide G_4_C_2_ repeat expansion in the *C9ORF72* gene accounts for almost 90% of the families presenting with both FTD and ALS symptoms (referred to as C9FTD/ALS; DeJesus-Hernandez et al., 2011; Renton et al., 2011). Patients can be mosaic for repeat size and often have longer repeats in brain tissue than in DNA isolated from blood samples (Fournier et al., 2019; Nordin et al., 2015; van Blitterswijk et al., 2013). So far, repeat sizes of 24-4400 have been reported (Iacoangeli et al., 2019; Van Mossevelde et al., 2017). Associations between repeat size and clinical diagnosis have not resulted in a clear picture (Fournier et al., 2019; Gijselinck et al., 2016; van Blitterswijk et al., 2013). Thus, the exact repeat size that triggers disease onset is not known.

Three mechanisms for the *C9ORF72* repeat expansion have been proposed to cause C9FTD/ALS (Balendra and Isaacs, 2018). First, hypermethylation of the repeat and surrounding CpG islands can lead to reduced levels of the normal C9ORF72 protein (Belzil et al., 2013; Waite et al., 2014). *C9orf72* knockout mice have shown its essential function in immunity, but do not present with FTD or ALS symptoms (Balendra and Isaacs, 2018). However, haploinsufficiency can still modify the effects of gain-of-function mechanisms via the normal cellular function of the C9ORF72 protein in autophagy and lysosomal biogenesis (Sellier et al., 2016; Shi et al., 2018). Second, repeat-containing RNA from both sense and antisense directions can form secondary structures (Kovanda et al., 2015; Su et al., 2014) and RNA foci (Gendron et al., 2013; Mizielinska et al., 2013). Repeat-containing RNA or RNA foci can sequester RNA-binding proteins and prevent their normal functioning in the cell (Haeusler et al., 2016). Third, the G_4_C_2_ repeat can also be translated into dipeptide repeats (DPRs) via repeat-associated non-ATG (RAN) translation (Ash et al., 2013; Gendron et al., 2013; Mori et al., 2013). RAN translation occurs in all reading frames of sense and antisense transcripts and results in the formation of poly-glycine-alanine (GA), poly-glycine-proline (GP), poly-glycine-arginine (GR), poly-proline-alanine (PA) and poly-proline-arginine (PR). DPRs have been found throughout the brains of C9FTD/ALS patients (Mackenzie et al., 2015) and poly-GR has particularly been associated with neurodegeneration (Gittings et al., 2020; Saberi et al., 2018; Sakae et al., 2018). Multiple cell and animal models have indicated the detrimental effect of the expression of both arginine-containing DPRs, poly-GR and poly-PR, and the slightly less toxic poly-GA (Balendra and Isaacs, 2018; Boeynaems et al., 2016; Jovicic et al., 2015; Kanekura et al., 2016; Kwon et al., 2014; Mizielinska et al., 2014; Swaminathan et al., 2018; Tao et al., 2015; Wen et al., 2014; Yamakawa et al., 2015; Yang et al., 2015). So far, 11 loss-of-function and ten gain-of-function *C9ORF72* mouse models have been published (reviewed by Balendra and Isaacs, 2018; Batra and Lee, 2017), as well as five DPR-only mouse models investigating the role of poly-GA (Schludi et al., 2017; Zhang et al., 2016), poly-GR (Zhang et al., 2018) and poly-PR (Hao et al., 2019; Zhang et al., 2019). All mouse models support a gain-of-function hypothesis in C9FTD/ALS, although not all BAC mice show neurodegeneration or a motor phenotype associated with ALS (reviewed by Balendra and Isaacs, 2018; Batra and Lee, 2017). The effect of DPRs has been studied extensively (reviewed by Balendra and Isaacs, 2018), but possible reversibility and the exact number of repeats needed for RAN translation *in vivo* have yet to be determined (Cleary et al., 2018; Green et al., 2016).

Here, we describe a new mouse model that expresses human *C9ORF72* 36× pure G_4_C_2_ repeats with 100-bp upstream and downstream human flanking regions under the expression of an inducible Tet-on promoter. This system allows for temporal and spatial expression of the repeat expansion. Expression of 36× pure G_4_C_2_ repeats was sufficient to produce sense DPRs and a locomotor phenotype upon 4 weeks of induction of expression. In order to study possible reversibility, expression was stopped after 1 or 2 weeks, followed by a washout period of 2-3 weeks to prevent further build-up and subsequent reduction of the amount of DPRs.

## RESULTS

### Generation and expression pattern of the human 36× G_4_C_2_ repeat mouse model

**Fig. 1.**
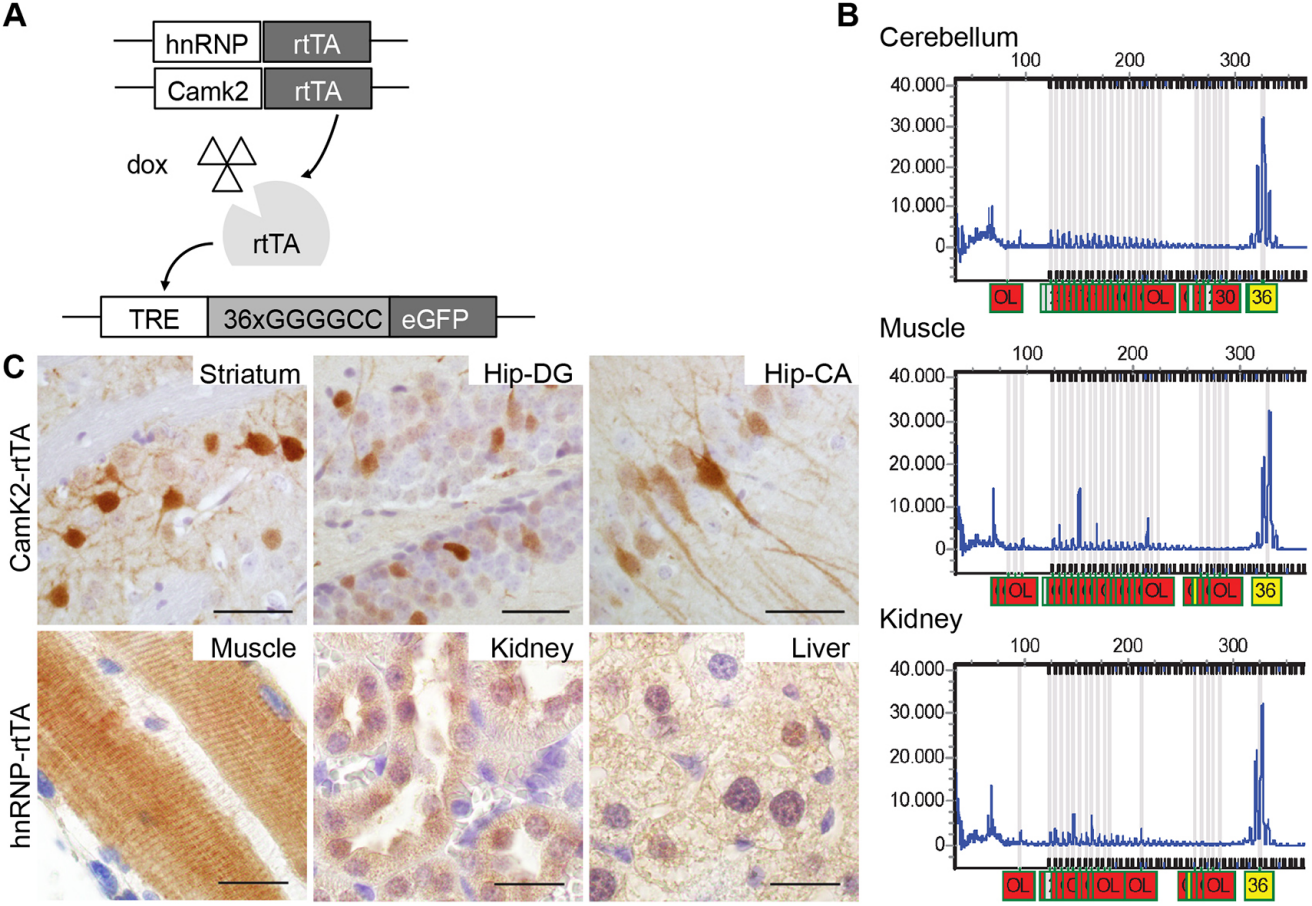
**Generation and expression of the 36**× **G_4_C_2_ repeat mouse model.** (A) Schematic of the Tet-on system. Mice either have a Camk2-alpha-rtTA or hnRNP-rtTA transgene that expresses rtTA in a brain-specific manner or in the whole body. Upon binding of dox, rtTA can bind the TRE response element and start transcription of the C9ORF72 G_4_C_2_ repeat expansion and GFP gene, which has its own start site. During the whole study, ST littermates were used as a control and received the same dox treatment. (B) DNA isolated from different tissues from the same mouse (17129-5 ladder mouse on dox for 4 weeks), and analyzed with an Asuragen C9ORF72 PCR kit, showed a repeat length of 36 in all tissues. This was repeated in at least three independent mice. OL, out of limit. (C) Upper panel: GFP expression was detected in striatum and hippocampus cornu ammonis and hippocampus dentate gyrus in DT Camk2-alpha-rtTA/TRE-36G_4_C_2_-GFP mice after dox administration. Lower panel: GFP expression in EDL muscle, kidney and liver of DT hnRNP-rtTA/TRE-36×G_4_C_2_-GFP mice. GFP staining was performed on all mice in this study. ST, 4 weeks dox, *n*=15; DT, 4 weeks dox, *n*=16. Scale bars: 20 µm.

We generated our mouse model from DNA isolated from the blood of a C9FTD patient and amplified the repeat in three consecutive PCR rounds using primers that flanked the *C9ORF72* repeat expansion (for primer sequences see Materials and Methods). The PCR product was cloned into a TOPO vector and subsequently into a Tet-on vector with a GFP reporter gene (Hukema et al., 2014) (Fig. 1A). Sequencing of this construct revealed a repeat size of 36× pure G_4_C_2_ repeats with 118-bp upstream and 115-bp downstream human flanking regions (Fig. S1). The transgene (containing the TRE promoter, 36× G_4_C_2_ repeats and the GFP gene) was injected into pronuclei of C57BL/6J mice. Founder mice were screened for the presence and size of the transgene and transmission to their offspring (*n*=3 lines containing the repeat, 1 line with expression). Genotyping for transgene presence was performed with primers located upstream of the repeat. Repeat size estimation was established using the Asuragen *C9ORF72* repeat kit that is also used in routine human diagnostics. Repeat size remained stable between generations and between multiple organs of the same mouse (Fig. 1B). Transgenic mice were born at Mendelian frequencies and showed normal viability.

Heterozygous transgenic mice containing the TRE-36×G_4_C_2_-GFP construct were bred with two different heterozygous rtTA driver lines. We chose the CamK2α (Ca^2+^/calmodulin-dependent protein kinase II linked reverse tetracycline-controlled transactivator) rtTA driver because of its validated expression in the hippocampus and cortex, brain areas that exhibit pathology in C9FTD/ALS patients (Odeh et al., 2011). To study expression in the rest of the body, we used an hnRNP (heterogeneous nuclear ribonucleoprotein 2B1) rtTA driver. The resulting litters consisted of four different genotypes referred to in the rest of the paper as double transgenic (containing both the TRE-36×G_4_C_2_-GFP construct and one of the rtTA constructs) or single transgenic littermates (containing only the TRE or only an rtTA-driver construct). Wild-type littermates were not used in this study. Mice were administered doxycycline (dox) in their drinking water at 6 weeks of age to turn on transgene expression, which revealed specific expression of GFP in the double transgenic (DT) mice only and no transgene expression in single transgenic (ST) mice (Fig. S2). Using the hnRNP-rtTA driver, we observed expression in almost all tissues, including extensor digitorum longus (EDL) muscle, liver, kidney (Fig. 1C) heart and lung (Fig. S2), but not in brain and spinal cord after a maximum of 4 weeks of dox treatment (Fig. S2). Using the Camk2-alpha-rtTA driver, we observed GFP expression only in striatum and hippocampus dentate gyrus and cornu ammonis, as expected (Fig. 1C). GFP expression was detectable after 1 week of dox administration and remained detectable over 6 months (data not shown).

### Human 36× G_4_C_2_ repeat mice show DPR expression but no RNA foci

**Fig. 2.**
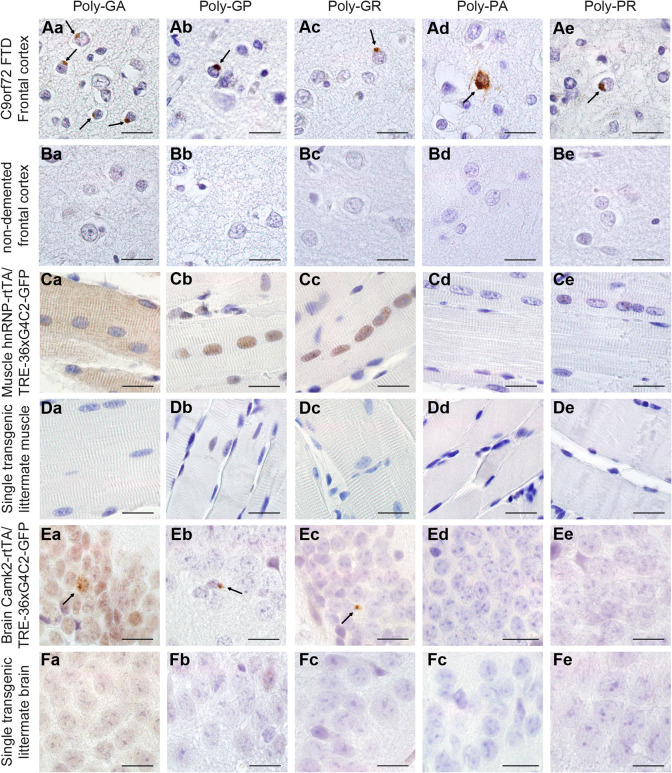
**Expression of 36**× **G_4_C_2_ human repeats is sufficient to evoke sense DPR formation *in vivo*.** (A,B) Human prefrontal cortex of C9FTD patients (A) or non-demented controls (B) were used as positive and negative controls for the detection of DPR pathology. Arrows indicate perinuclear aggregates of DPRs. (C) In TRE-36×G_4_C_2_-GFP/hnRNP-rtTA DT mice, poly-GA showed both diffuse cytoplasmic and nuclear localization, whereas diffuse poly-GP and poly-GR were observed in the nucleus of the EDL muscle. (D,F) ST littermates received the same dox treatment and contained only one transgene, either TRE only or rtTA only, and were all negative for DPRs. (E) TRE-36×G_4_C_2_-GFP/Camk2-alpha-rtTA DT mice showed some sparse perinuclear aggregates of sense DPRs in the hippocampus dentate gyrus (indicated by arrows). DPR staining was performed on all mice in this study. ST, 4 weeks dox, *n*=15; DT, 4 weeks dox, *n*=16. Scale bars: 20 µm.

To further characterize the expression of the transgene in our mouse model, we performed fluorescence *in situ* hybridization (FISH) to test for the presence of sense and antisense RNA foci. Unfortunately, we were unable to detect RNA foci in multiple organs at 1-24 weeks of age in any of the driver lines (Fig. S3), despite the fact that our protocol was optimized to detect RNA foci in post-mortem human C9FTD/ALS frontal cortex paraffin tissue used as a positive control (Fig. S3). Sense-transcribed DPRs (poly-GA, -GP and -GR) were present in all GFP^+^ tissues of DT hnRNP-rtTA mice (Fig. 2). We did not observe antisense DPRs in mice (Fig. 2C-F), although we could detect them in C9FTD/ALS patient frontal cortex sections that were used as a positive control (Fig. 2A,B). To validate that no antisense transcripts were produced in our mouse model, we performed RT-PCR (Fig. S4). Again, antisense transcripts were only observed in RNA isolated from frozen frontal cortex of a human C9FTD patient (Fig. S4).

Interestingly, sense DPRs differed in subcellular localization. Poly-GA was visible as diffuse nuclear and cytoplasmic labeling in DT hnRNP-rtTA mice, whereas poly-GP and -GR were only observed in the nucleus (Fig. 2C,D; Fig. S5). In the DT Camk2-alpha-rtTA mice, we could detect some small perinuclear aggregates in the striatum and hippocampus after 24 weeks of dox administration (Fig. 2E,F). However, the numbers of aggregates were very rare (about one aggregate per sagittal brain section). Longer follow-up of these mice is not possible, as administration of dox for more than 6 months often leads to intestinal problems and an unacceptable level of discomfort for the mice. Both Camk2- and hnRNP-driven 36×G_4_C_2_ repeat mice showed no abundant pathological hallmarks of C9FTD/ALS, including p62 and phosphorylated TAR DNA-binding protein (pTDP-43) aggregates in brain and muscle (Fig. S6). Additionally, we did not observe any signs of neurodegeneration (cleaved caspase-3 staining, Fig. S6), astrogliosis or microgliosis (Fig. S7). As DPR inclusions were very rare in Camk2-alpha-rtTA mice and expression of DPRs was evident in the hnRNP-rtTA mice, we chose to focus on the DT hnRNP-rtTA mice for further assessment of the toxic effect of DPR expression in multiple organs *in vivo*.

### The 36× G_4_C_2_ repeat mice develop a locomotor phenotype, rapid muscular dystrophy and neuromuscular junction abnormalities

**Fig. 3.**
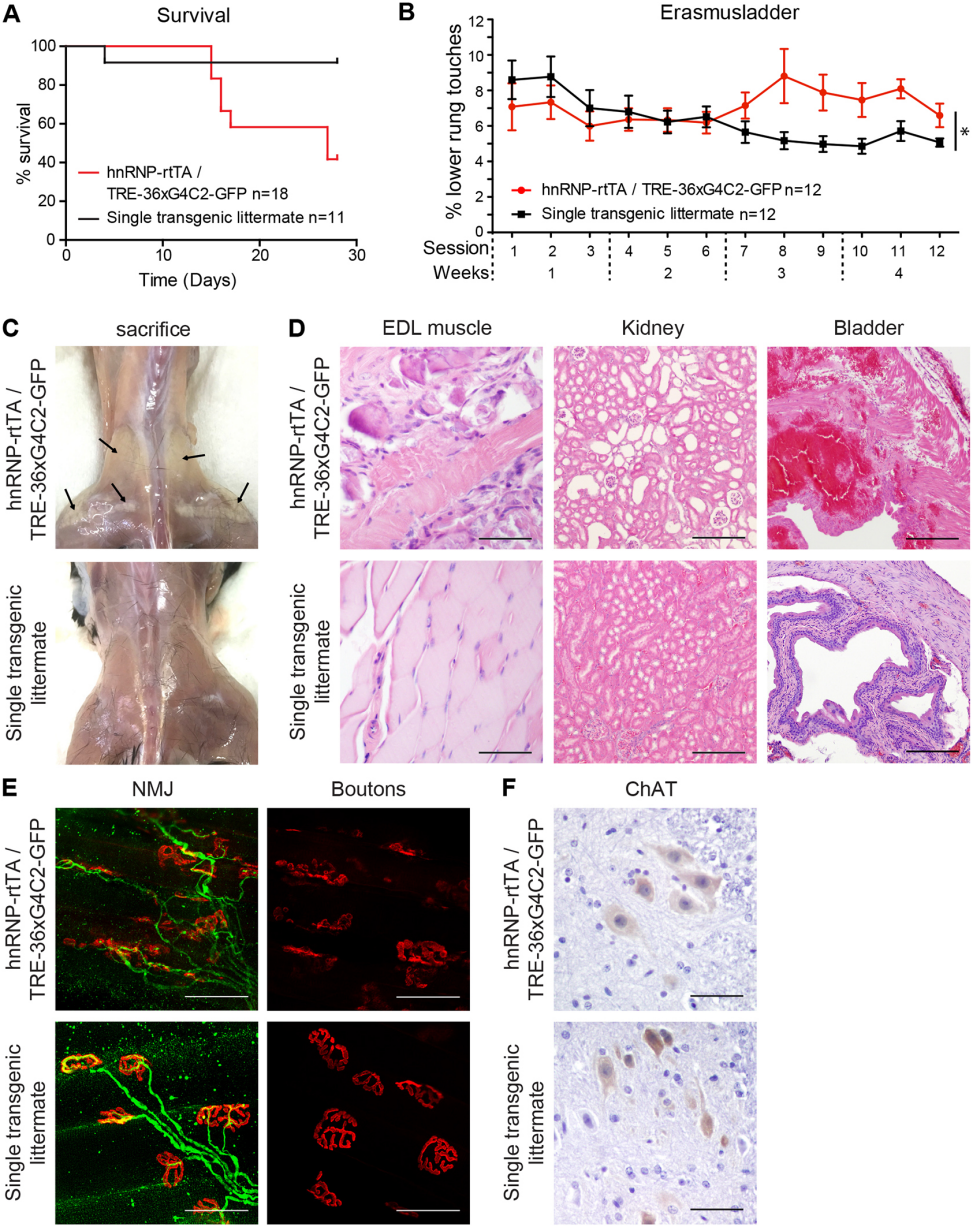
**Expression of 36**× **G_4_C_2_ human repeats *in vivo* causes a locomotor phenotype and muscular dystrophy within 4 weeks.** (A) TRE-36×G_4_C_2_-GFP/hnRNP-rtTA DT mice that receive dox showed reduced survival after 1-3 weeks (*n*=18) compared to ST littermates [containing only one transgene (either TRE only or rtTA only)] who received the same dox treatment (*n*=11). (B) Mice that survive developed a locomotor phenotype on the Erasmus ladder after 7 sessions (3 sessions/week). *N*=12 mice per group. Note that from session 7 onwards, some mice had to be excluded due to severe pathology or incapability to cope with the behavioral assay. Session 12 includes data from *N*=10 ST control and *N*=8 DT mice. All mice received the same dox treatment. *P=0.0001 for genotype and *P*<0.0001 for the interaction between genotype and time (two-way ANOVA). Data are mean±s.e.m. (C) When sacrificed, DT mice exhibited back and upper leg muscles with a white appearance (indicated by arrows). (D) H&E staining of the EDL muscle, kidney and bladder of DT mice. (E) NMJ staining of the EDL muscle showed dissolving boutons (red, α-bungarotoxin) and disorganized axonal projections (green, neurofilament antibody). (F) The number of ChAT^+^ motor neurons in the spinal cord did not differ between DT and ST control littermates. All stainings were performed on all mice in this study. ST, 4 weeks dox, *n*=15; DT, 4 weeks dox, *n*=16. Scale bars: 50 µm (D, EDL and kidney images); 200 µm (D, bladder images); 50 µm (E); 20 µm (F).

Expression of 36× G_4_C_2_ repeats using the hnRNP-rtTA driver led to profound toxicity. We started with dox treatment in 6-week-old mice to avoid DPRs affecting normal development, which would complicate behavioral and functional read-out. A large proportion (45%) of DT mice quickly declined in body weight in the first 2-3 weeks after dox administration and had to be sacrificed (Fig. 3A). Mice that quickly lost weight after 2.5 weeks showed general sickness symptoms (weight loss, bad condition of the fur, reduced activity and shivering) and an enlarged bladder. The majority of mice survived longer and did not lose weight but developed a locomotor phenotype on the Erasmus ladder (Fig. 3B; Fig. S8). This is a locomotor test based upon a horizontal ladder with alternating higher and lower rungs. Healthy C57Bl6/J mice prefer to walk on the higher rungs and avoid touching the lower rungs (Vinueza Veloz et al., 2015). DT mice began to touch the lower rungs more often after 2 weeks of dox treatment, although their performance was initially comparable to that of their ST littermates (Fig. 3B; Fig. S8; two-way ANOVA analysis: *P*=0.0001 for genotype and *P*<0.0001 for the interaction between genotype and time). DT mice sacrificed after 4 weeks of dox treatment displayed a white appearance of leg and back muscles macroscopically (Fig. 3C). At the histological level, a massive distortion of muscle fibers could be observed (Fig. 3D). Histological analysis of other tissues revealed enlarged renal tubules in the kidney and hemorrhages in the bladder of mice that quickly lost weight at 2.5 weeks (Fig. 3D). Analyses of the neuromuscular junctions (NMJs) by whole-mount immunostaining of the EDL muscle showed distortion of the muscular boutons and projecting motor neuron axons after 4 weeks of dox treatment (Fig. 3E). The number of motor neurons assessed by choline acetyltransferase (ChAT) staining of the spinal cord was not different between DT mice and ST control littermates (Fig. 3F). Together, these data indicate that expression of 36 pure G_4_C_2_ repeats in the body (but not expression in the brain) in our mouse model causes multisystem dysfunction, including urinary system problems and muscular dystrophy over the time course of 1 month.

### Early withdrawal of 36× G_4_C_2_ repeat expression can prevent but not reverse the muscular dystrophy phenotype

**Fig. 4.**
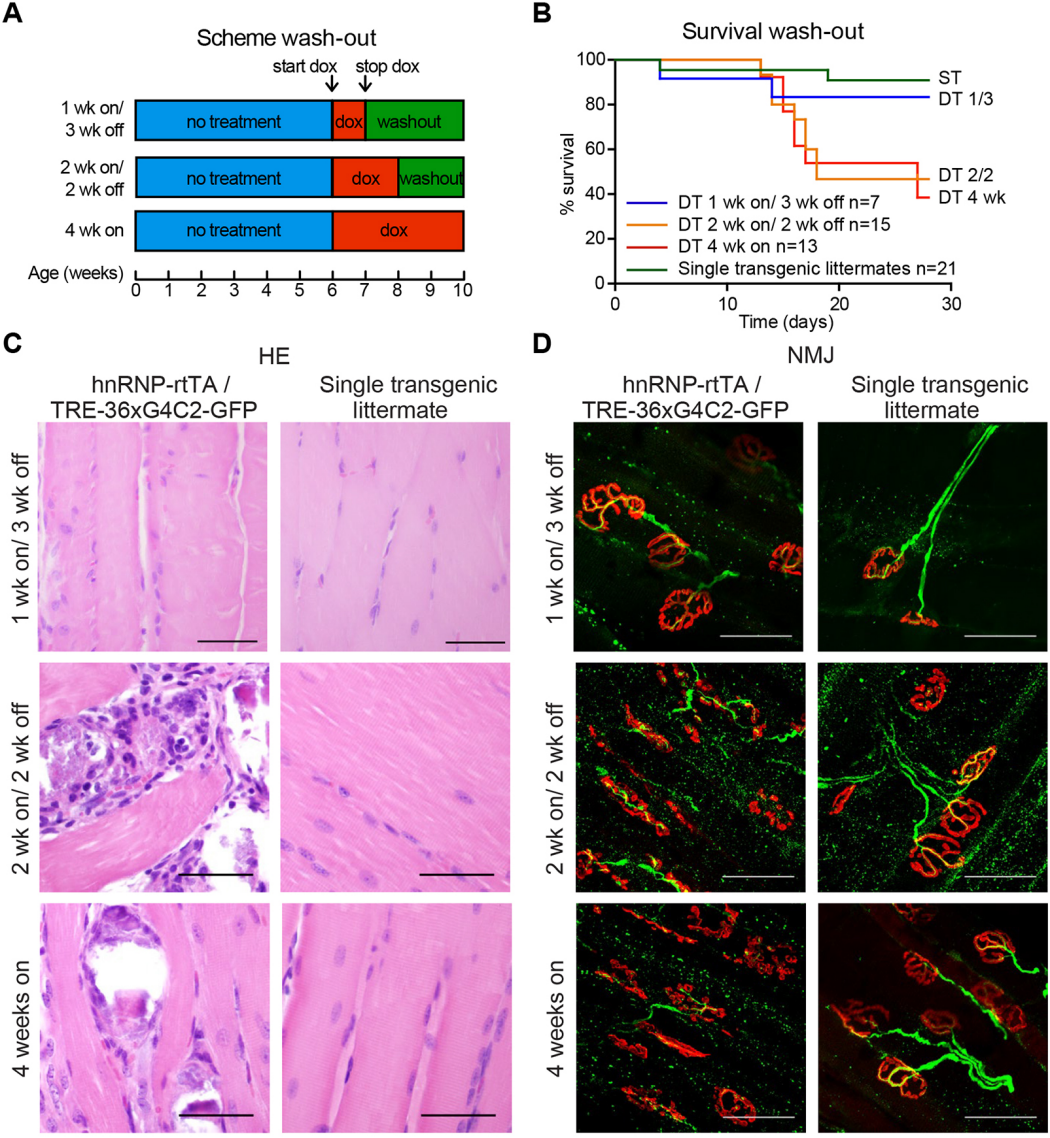
**Early dox withdrawal can prevent but not reverse the muscular dystrophy and NMJ phenotype of TRE-36×G4C2-GFP/hnRNP-rtTA DT mice.** (A) Schematic of different washout groups. (B) Survival curve for the washout experiment. Mice that received 1 week dox followed by 3 weeks washout (1 week on/ 3 weeks off, *n*=7) showed high survival. Two weeks dox followed by 2 weeks washout (2 weeks on/ 2 weeks off, *n*=15) showed the same reduction in survival as 4 weeks continuous dox administration (4 weeks on, *n*=13). ST littermates [containing only one transgene (either TRE only or rtTA only)] were distributed over the groups and received the same dox treatment (*n*=21). (C) H&E staining of the EDL muscle remained normal in the 1 week on/ 3 weeks off group but showed distortion in TRE-36×G_4_C_2_-GFP/hnRNP-rtTA DT mice that received 2 weeks dox followed by 2 weeks of normal drinking water. (D) NMJ of the EDL muscle showed collapsed boutons (red, α-bungarotoxin) and axonal projections (green, neurofilament antibody) in DT groups that received 2 or more weeks of dox. All stainings were performed on all mice in this study. Numbers per group were: ST, 1 week dox, *n*=7; DT, 1 week dox, *n*=8; ST, 1 week on/3 weeks off, *n*=4; DT, 1 week on/3 weeks off, *n*=7; ST, 2 weeks dox, *n*=6; DT, 2 weeks dox, *n*=8; ST, 2 weeks on/2 weeks off, *n*=6; DT, 2 weeks on/2 weeks off, *n*=5; ST, 4 weeks dox, *n*=15; DT, 4 weeks dox, *n*=16. Scale bars: 50 µm.

**Fig. 5.**
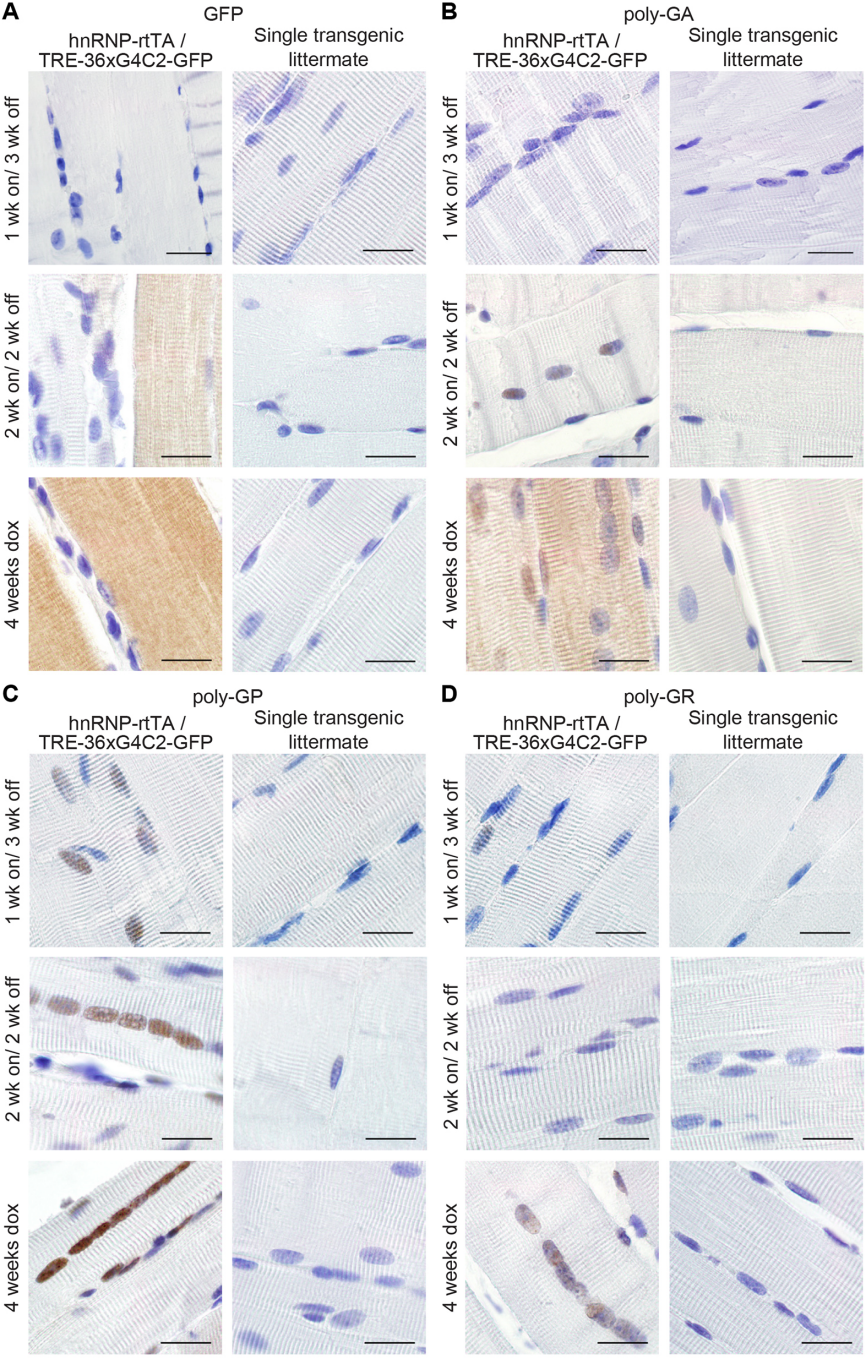
**GFP and sense DPRs are reduced after dox withdrawal.** (A) GFP staining of EDL muscle of TRE-36×G_4_C_2_-GFP/hnRNP-rtTA DT mice showed a reduction in the intensity of staining when mice received 1 or 2 weeks of dox water, followed by 2-3 weeks of normal drinking water compared to DT littermates that received 4 weeks of dox. (B) Poly-GA staining of EDL muscle showed clearance of cytoplasmic poly-GA in all washout groups but retention of nuclear poly-GA after 2 weeks of dox withdrawal. (C,D) Poly-GP staining is reduced in the nucleus of EDL muscle (C) and Poly-GR staining is cleared from nuclei of EDL muscles after dox withdrawal (D). ST littermates, consisting of either TRE only or rtTA only, received the same dox treatment and were all negative for GFP and DPRs. All stainings were performed on all mice in this study. Numbers per group were: ST, 1 week dox, *n*=7; DT, 1 week dox, *n*=8; ST, 1 week on/3 weeks off, *n*=4; DT, 1 week on/3 weeks off, *n*=7; ST, 2 weeks dox, *n*=6, DT, 2 weeks dox, *n*=8; ST, 2 weeks on/2 weeks off, *n*=6; DT, 2 weeks on/2 weeks off, *n*=5; ST, 4 weeks dox, *n*=15; DT, 4 weeks dox, *n*=16. Scale bars: 20 µm.

**Fig. 6.**
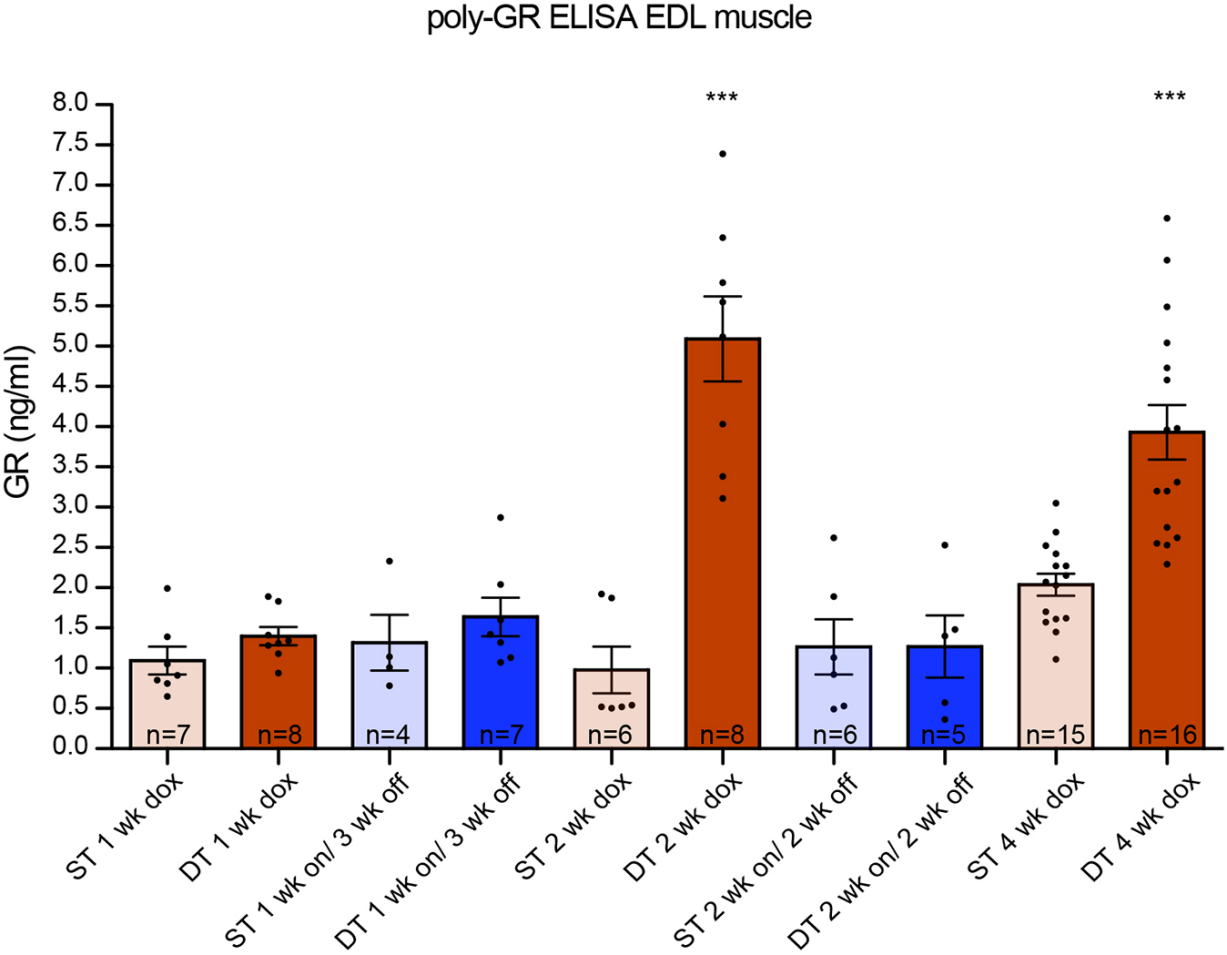
**Expression of 36× G_4_C_2_ human repeats *in vivo* generates high levels of poly-GR, which are reduced to ST level after dox withdrawal.** ELISA assessing poly-GR levels from mouse EDL muscle. Poly-GR was detectable in TRE-36×G_4_C_2_-GFP/hnRNP-rtTA DT mice that received 2-4 weeks of dox. DT mice that received 1-2 weeks dox followed by 2-3 weeks washout have reduced amounts of poly-GR, similar to ST levels. A one-way ANOVA (***P=0.0001) Bonferroni post test shows that the DT 2 weeks dox and DT 4 weeks dox groups were significantly different from all the other groups, but not from each other. All other ST and DT washout groups were not significantly different from each other. ST littermates [consisting of only one transgene (either TRE only or rtTA only)] received a similar dox treatment as DT animals. Numbers per group were: ST, 1 week dox, *n*=7; DT, 1 week dox, *n*=8; ST, 1 week on/3 weeks off, *n*=4; DT, 1 week on/3 weeks off, *n*=7; ST, 2 weeks dox, *n*=6; DT, 2 weeks dox, *n*=8; ST, 2 weeks on/2 weeks off, *n*=6; DT, 2 weeks on/2 weeks off, *n*=5; ST, 4 weeks dox, *n*=15; DT, 4 weeks dox, *n*=16. Data are mean±s.e.m.

In order to investigate whether the phenotype could be reversed, we administered dox to 6-week-old DT and ST mice for 1 or 2 weeks and then changed them back to normal drinking water for 3 and 2 weeks, respectively (washout scheme shown in Fig. 4A). Mice that only received 1 week of dox followed by 3 weeks of washout showed high survival rates and normal muscle and NMJ integrity (Fig. 4). Approximately half of the DT mice that received 2 weeks of dox followed by 2 weeks of washout showed a rapid reduction in body weight after 2-3 weeks and did not survive to the end of the experiment (Fig. 4B). This washout group was indistinguishable from the 4 weeks dox DT group, with regards to survival, muscle and NMJ integrity. Haematoxylin and eosin (H&E) staining showed that parts of the EDL displayed abnormal organization (Fig. 4C), and the NMJ showed disrupted boutons and axonal projections (Fig. 4D). Immunostaining for GFP, poly-GA and -GP in muscles of both washout groups showed a clear reduction in their levels but poly-GA and -GP were still detectable in nuclei of the 2 weeks on/ 2 weeks off group (Fig. 5). Only poly-GR could not be detected anymore (Fig. 5). In the kidney, GFP and all sense DPRs were cleared efficiently after dox withdrawal (Fig. S9). This indicates that DPR clearance is different for each organ or cell type. To study build-up and reduction of DPRs in a more quantitative way, we developed an ELISA for poly-GR, the DPR that shows the highest reported cellular toxicity. Poly-GR levels in EDL muscle increased significantly between the first and second week of dox administration, after which they stayed high (Fig. 6). In contrast, poly-GR levels in mice from the reversibility groups were reduced to similar levels as ST control mice (one-way ANOVA test: *P*=0.0001 with Bonferroni post test; Fig. 6). Together, our data show that early withdrawal of 36× pure G_4_C_2_ repeat expression can prevent the accumulation of DPRs and the concurrent phenotype. Expression of 36× pure G_4_C_2_ repeat expression for 2 weeks and subsequent withdrawal for 2 weeks is not sufficient to completely clear DPRs and reverse muscular dystrophy *in vivo*.

## DISCUSSION

In this study, we demonstrate that expression of 36× pure G_4_C_2_ repeats *in vivo* is sufficient to cause NMJ abnormalities and muscular dystrophy, leading to a specific locomotor phenotype within 4 weeks of transgene expression. Expression for 4 weeks did not evoke DPR expression in the brain, probably because 4 weeks are not long enough for build-up of DPRs and for dox to pass the blood-brain barrier (Michel et al., 1984). Expression of 36× pure G_4_C_2_ repeats for 24 weeks in the murine brain, using a Camk2-alpha-rtTA driver, was also not sufficient to result in pathology or neurodegeneration, making this mouse model inadequate for studying brain-specific DPR toxicity. We speculate that expression levels of the 36× G_4_C_2_ repeat RNA and DPRs in our Camk2-alpha-rtTA driven model are not high enough to induce neuropathology. Alternatively, the repeat length might not be long enough to evoke neurodegeneration. Other gain-of-function mouse models did show sense DPR pathology and a cognitive phenotype upon (over)expression of longer repeats (Chew et al., 2015; Herranz-Martin et al., 2017; Jiang et al., 2016; Liu et al., 2016). Mice expressing 500 repeats show a more severe phenotype compared with 29/36 repeat mice (Liu et al., 2016).

Upon expression of 36× pure G_4_C_2_ repeats in the body, our mouse model shows rapid muscular dystrophy and a locomotor phenotype. The phenotype could be caused by NMJ abnormalities or skeletal muscle dysfunction. Interestingly, DPR pathology has recently been found in skeletal muscle of C9ALS patients (Cykowski et al., 2019). DPR pathology has not been reported in human in tissues such as the kidney and bladder, even though C9ORF72 is expressed in these organs and C9KO mice show immune-mediated kidney damage (Atanasio et al., 2016; DeJesus-Hernandez et al., 2011). The pathology in our mouse model could be evoked by the relative rapid and strong repeat expression compared to the lower expression levels observed in C9FTD/ALS patients, but it would be interesting to investigate how widespread DPR pathology is. Many *C9ORF72* mouse models lack locomotor symptoms due to unknown factors (Jiang et al., 2016; O'Rourke et al., 2015; Peters et al., 2015), and NMJ abnormalities have only been described in one BAC mouse model and one AAV-102x interrupted G_4_C_2_ mouse model (Herranz-Martin et al., 2017; Liu et al., 2016). Our mouse model shows similarities to the BAC 29/36 repeat mouse model reported by Liu et al. (2016), as both models show DPR pathology but no RNA foci. However, the phenotype in our mouse model develops faster (within 4 weeks after dox administration) than reported by Liu et al. (2016) (first symptoms started after 16 weeks of age). Differences in disease onset might be due to differences in expression levels. For example, lack of a phenotype was observed in a 37 repeat mouse with low expression levels (Liu et al., 2016), whereas our 36 repeat and the BAC 29/36 repeat mouse with higher expression levels clearly show a phenotype.

So far, the minimum repeat size to evoke RNA foci and DPR formation *in vivo* has remained unknown. A BAC mouse model of 110 repeats did not contain any RNA foci (Jiang et al., 2016), whereas BAC mice with longer repeat sizes did present with RNA foci (Jiang et al., 2016; Liu et al., 2016; O'Rourke et al., 2015; Peters et al., 2015). On the other hand, AAV-mediated overexpression of 10 or 66 repeats did evoke RNA foci (Chew et al., 2015; Herranz-Martin et al., 2017), indicating that formation of RNA foci could also depend on expression levels. Even though we did not detect any RNA foci in our 36× repeat mouse model, we cannot exclude an effect of repeat RNA on the observed phenotype. Repeat-containing RNA molecules might still be able to sequester molecules or proteins and affect their normal function of cellular processes.

Sense DPRs were detected as diffuse cytoplasmic or nuclear staining and did not form aggregates in our hnRNP-driven mouse model. Recent publications on poly-GR and -PR mouse models suggest that soluble poly-GR and -PR are sufficient to cause neurodegeneration and behavioral deficits (Zhang et al., 2018, 2019). For poly-GA, aggregation seems necessary for its toxicity (Zhang et al., 2016). Thus, DPRs might differ in their abilities to aggregate, which can change their molecular targets and their effects on several cellular compartments and functions. Interestingly, poly-GA can spread throughout the brain and influence the aggregation of poly-GR and -PR (Darling et al., 2019; Morón-Oset et al., 2019; Yang et al., 2015), and this has been confirmed in AAV-66 and AAV-149x mice, in which poly-GA and -GR co-aggregate in cells with poly-GA aggregates, but poly-GR remains diffuse in cells devoid of poly-GA (Chew et al., 2019; Zhang et al., 2018). Poly-GA expression can even partially suppress poly-GR-induced cell loss at the wing in a *Drosophila* model (Yang et al., 2015). Co-overexpression of poly-GA also abolished cellular toxicity of low concentrations of poly-PR in NSC34 cells (Darling et al., 2019). Other interactions between DPRs are still unknown and need further investigation. We did not detect antisense DPRs in our mouse model, perhaps because antisense C_4_G_2_ RNA is not transcribed or antisense DPR levels might be too low to detect.

Another point of interest is the lack of apparent pTDP-43 pathology in multiple *C9ORF72* mouse models. TDP-43 pathology is thought to be a late event in the pathogenesis of C9FTD/ALS (Balendra and Isaacs, 2018). Several mouse models already show behavioral phenotypes and some mild neurodegeneration before the onset of pTDP-43 neuropathology (Herranz-Martin et al., 2017; Jiang et al., 2016; Schludi et al., 2017; Zhang et al., 2018, 2016). Changes in pTDP-43 solubility or cellular localization could already arise and contribute to cellular distress without the formation of cytoplasmic aggregates per se (Lee et al., 2019). Indeed, several reports of C9FTD/ALS cases showed affected individuals with DPR pathology but mild or absent TDP-43 pathology (Baborie et al., 2015; Gijselinck et al., 2012; Mori et al., 2013; Proudfoot et al., 2014; Vatsavayai et al., 2016). Together, our hnRNP-driven mouse model shows that expression of diffuse labeled sense DPRs is sufficient to cause cellular toxicity without the need for RNA foci and pTDP-43 pathology.

The rapid translation of current knowledge into therapeutic intervention studies requires robust *in vivo* drug discovery screens (Jiang and Ravits, 2019). So far, antisense oligonucleotide (AON) therapy has been tested in a BAC mouse model for *C9ORF72*, and successfully reduced the amount of RNA foci and DPRs (Jiang et al., 2016). However, it remains unknown whether this AON can also reduce motor symptoms associated with the *C9ORF72* repeat. New therapies are under development, including small molecules targeting RAN translation (Green et al., 2019; Hu et al., 2017; Kramer et al., 2016; Simone et al., 2018; Su et al., 2014; Zamiri et al., 2014; Zhang et al., 2015) and antibody therapy against poly-GA (Nguyen et al., 2020; Zhou et al., 2019). These therapies can be easily tested in our mouse model, as it develops a quick and robust phenotype. Our mouse model can be used as proof of principle for whole-body toxicity of DPRs. We demonstrated that 1 week of expression followed by 3 weeks of washout (expression turned off) prevented the accumulation of DPRs and the associated cellular toxicity. However, 2 weeks of expression followed by 2 weeks of washout is not sufficient to prevent mice from developing muscular dystrophy. This indicates that transgene RNA or DPRs that were already produced during the first 2 weeks of dox administration continue to exercise their toxic effects. A recent publication estimated the half-lives of most DPRs to be >200 h (Westergard et al., 2019). The half-life was longer for poly-GA puncta than for diffuse poly-GA, and increased for poly-GR when localized in the nucleus (Westergard et al., 2019). Interestingly, poly-GP remains detectable after 1 week on/3 weeks off dox, indicating that poly-GP is not turning over as quickly as the other DPRs. Still, the animals of this reversibility group are improving, suggesting a bigger impact on the toxicity of poly-GA and poly-GR, as shown previously (Balendra and Isaacs, 2018; Mizielinska et al., 2014). In general, earlier intervention might be able to halt or reverse symptoms, but the preferred time window for treatment is probably before the onset of symptoms.

Together, we provide evidence that the expression of human 36× pure G_4_C_2_ repeats is sufficient to evoke RAN translation and a locomotor phenotype *in vivo*. High expression of sense DPRs driven by hnRNP-rtTA caused rapid progression of muscular dystrophy and NMJ disruption. This mouse model allows for fast *in vivo* screening of new drugs and compounds that act on the systemic toxicity of sense DPRs.

## MATERIALS AND METHODS

### Cloning

DNA obtained from C9FTD patient 09D-5781 was assessed for the *C9ORF72* repeat expansion with an Asuragen kit used according to manufacturer's protocol, and contained at least 54 repeats. DNA was amplified in three consecutive rounds of PCR with primers flanking the *C9ORF72* repeat expansion (forward primer, 5′-CCACGGAGGGATGTTCTTTA-3′ and reverse primer, 5′-GAAACCAGACCCAAACACAGA-3′) and a PCR mix containing 50% betaine. The PCR program started with 10 min at 98°C, followed by 35 cycles of 35 s at 98°C, 35 s at 58°C and 3 min at 72°C and finished with 10 min at 72°C. The PCR product was cloned into TOPO vector PCR2.1, and restriction analysis with BsiEI (New England Biolabs) for 1 h at 60°C revealed a G_4_C_2_ repeat expansion estimated to consist of ∼50 repeats. Next, the TRE-90CGG-GFP vector (Hukema et al., 2014) was restricted with SacII (New England Biolabs), and the 90xCGG repeat expansion was replaced with the 50x G_4_C_2_ repeat expansion. This vector was sequenced using a primer in the TRE sequence (5′-CGGGTCCAGTAGGCGTGTAC-3′) and revealed a repeat expansion of 36× G_4_C_2_. The final vector was cut with AatII, PvuI and NdeI (NEB), and the band containing the TRE-36× G_4_C_2_-GFP construct was isolated from a gel, dissolved in injection buffer (10 mM Tris-HCl, pH 7.4, 0.25 mM EDTA) and used to generate transgenic mice. Experiments on human material were performed under informed consent and approved by the Medical Ethical Test Committee (METC). All investigations with human materials were conducted according to the principles expressed in the Declaration of Helsinki.

### Animals

Pronuclei from oocytes of C57BL/6JRj wild-type mice were injected to create a new transgenic line harboring the TRE-36×G_4_C_2_-GFP construct. Genotyping was performed using primers located in the 5′ region of the repeat expansion (forward, 5′-GGTACCCGGGTCGAGGTAGG-3′ and reverse, 5′-CTACAGGCTGCGGTTGTTTCC-3′). Founder mice, F1 and F2, were screened in an animal welfare assessment by the local animal caretakers and scored as normal for litter size and health characteristics. All mice were housed in groups of two to four and were allowed to have free access to standard laboratory food and water. They were kept in a 12-h light/dark cycle. TRE-36×G_4_C_2_-GFP mice were crossed with hnRNP-rtTA (Katsantoni et al., 2007) or Camk2-alpha-rtTA (kind gift of Rob Berman, The University of California, Davis, USA) on a C57BL/6JRj wild-type background. Offspring should include 25% of DT mice (harboring both the TRE and one of the rtTA constructs), 50% of ST littermates (harboring either the TRE or the rtTA construct) and 25% of wild-type littermates (having no transgene). At 6 weeks of age, mice of both sexes were exposed to dox (Sigma-Aldrich) (4 grams/l) combined with sucrose (50 grams/l) dissolved in drinking water. Both ST and DT mice received dox water. To monitor their health and wellbeing, mice were weighed every weekday while on dox water (data not shown). TRE-36×G_4_C_2_-GFP/hnRNP-rtTA mice were sacrificed by cervical dislocation after a maximum of 4 weeks of dox administration. The TRE-36×G_4_C_2_-GFP/Camk2-alpha-rtTA mice were sacrificed by cervical dislocation after a maximum of 24 weeks of dox administration. As required by Dutch legislation, all experiments were approved in advance by the institutional Animal Welfare Committee (Erasmus University Medical Center, Rotterdam, The Netherlands).

### Erasmus ladder

The Erasmus ladder (Noldus, Wageningen, The Netherlands) is a fully automated test for detecting motor performance in mice (Vinueza Veloz et al., 2012). It consists of a horizontal ladder between two shelters, which are equipped with a bright white LED spotlight and pressurized air outlets. These are used as cues for the departure from the shelter box to the other shelter box. The ladder has 2×37 rungs for the left and right side. All rungs have pressure sensors, which are continuously monitoring and registering the walking pattern of the mouse. The rungs are placed in an alternating high/low pattern. Wild-type C57Bl6 mice prefer to walk on the higher rungs, avoiding touching the lower rungs (Vinueza Veloz et al., 2015). The mouse was placed in the starting box and after a period varying from 9 to 11 s, the LED light turned on and the mouse was supposed to leave the box. If the mouse left the box before the light turned on a strong air flow drove the mouse back into the box, and the waiting period restarted. If the mouse did not leave the box within 3 s after the light turned on, a strong air flow drove the mouse out of the box. When the mouse arrived in the other box, the lights and air flow turned off and the waiting period from 9 to 11 s started and the cycle repeated again, making mice run back and forth on the ladder. Mice were trained on the Erasmus ladder at the age of 5 weeks, every day for 5 days. The mice were trained to walk the ladder for 42 runs each day. At the age of 6 weeks, the mice received dox/sucrose water and were tested on Monday, Wednesday and Friday on the Erasmus ladder. The average percentage of lower rung touches was calculated over 42 runs per session.

### NMJ staining

EDL muscles were fixed in 1% paraformaldehyde overnight. The muscles were washed in PBS and permeabilized in 2.5% Triton X-100 (Sigma-Aldrich) in PBS for 30 min and incubated in 1 µg/ml α-bungarotoxin-TRITC (Invitrogen) in 1 M NaCl for 30 min. Subsequently, muscles were incubated for 1 h in a blocking solution [4% bovine serum albumin (BSA), 0.5% Triton X-100]. After blocking, the muscles were incubated with a polyclonal chicken anti-neurofilament antibody (2BScientific) 1:500 in blocking solution overnight at 4°C, followed by incubation for 4 h with anti-chicken Alexa Fluor 488 antibody (Jackson ImmunoResearch). Finally, the muscles were mounted on slides with 1.8% low-melting point agarose (Thermo Fisher Scientific) and images were taken using a Zeiss LSM700 confocal microscope. The first author was blinded during image acquisition.

### Fluorescent *in situ* hybridization

Brain and EDL muscle tissues of mice were fixed in 4% paraformaldehyde overnight. Tissues were dehydrated and embedded in paraffin, and cut into 6 µm sections using a rotary microtome. Post-mortem human C9FTD/ALS frontal cortex paraffin tissue was used as a positive control for RNA foci detection. Sections were deparaffinized using xylene, and rehydrated in a standard alcohol series. Antigen retrieval was established in 0.01 M sodium citrate with pH 6 using microwave treatment of 1×9 min followed by 2×3 min at 800W. Subsequently, the slides were dehydrated in an alcohol series and briefly dried in air. Next, pre-hybridization was performed in hybridization solution (dextran sulphate 10% w/v, formamide 50%, 2× SSC) for 1 h at 65°C. After pre-hybridization, hexanucleotide sense oligo (5′-Cy5-4xGGGGCC-3′) and hexanucleotide antisense oligo (5′-Cy5-4xCCCCGG-3′) probes (IDT) were diluted to 40 nM in hybridization solution and heated to 95°C for 5 min. The slides were hybridized with probe mix overnight at 65°C. After hybridization, the slides were washed once with 2× SSC/0.1% Tween 20 and three times with 0.1× SSC at 65°C. Subsequently, slides were stained with Hoechst (Invitrogen), washed with PBS and stained with Sudan Black (Sigma-Aldrich). Finally, slides were dehydrated and mounted using Pro-Long Gold mounting solution (Invitrogen), and images were taken using a Zeiss LSM700 confocal microscope. C9FTD/ALS and non-demented control human brain sections were provided by the Dutch Brain Bank.

### Immunohistochemistry

Mouse tissues were fixed in 4% paraformaldehyde overnight, and dehydrated and embedded in paraffin. Sections (6 µm) were cut using a rotary microtome. Sections were deparaffinized using xylene and rehydrated in an alcohol series. Antigen retrieval was established in 0.01 M sodium citrate with pH 6 using microwave treatment of 1×9 min followed by 2×3 min at 800W. Endogenous peroxidase activity was blocked with 3% H_2_O_2_ and 1.25% sodium azide. Immunostaining was performed overnight at 4°C in PSB block buffer (1× PBS/0.5% protifar/0.15% glycine) and with the primary antibodies (see Table S1 for all antibodies used in this study). The next day, sections were washed with PBS block buffer, and antigen-antibody complexes were visualized by incubation with DAB substrate (Dako) after incubation with Brightvision poly-horseradish peroxidase (HRP)-linker (Immunologic) or anti-mouse/rabbit HRP (Dako). Slides were counterstained with Mayer's haematoxylin and mounted with Entellan (Merck Millipore). The slides were imaged using an Olympus BX40 microscope.

### Protein isolation

Before lysing, EDL muscle samples were thawed on ice and supplied with RIPA buffer containing 0.05% protease inhibitors (Roche) and 0.3% 1 M DTT (Invitrogen). Samples were mechanically lysed, followed by 30 min incubation on ice. After 30 min incubation, mechanical lysing was repeated and samples were centrifuged for 20 min at 17 ***g*** at 4°C, followed by 3×1 min sonication. After sonication, samples were centrifuged for 20 min at 17 ***g*** at 4°C and the supernatant was used for ELISA. Whole protein content was determined using a BCA assay (Thermo Fisher Scientific).

### ELISA

MaxiSorp 96-well F-bottom plates (Thermo Fisher Scientific) were coated for 2 h with 5 µg/ml monoclonal GR antibody, followed by overnight blocking with 1% BSA in PBS-Tween (0.05% Tween 20, Sigma-Aldrich) at 4°C. After washing, 300 µg total protein lysate was added per sample. A 15x GR synthetic peptide (LifeTein) was used as a positive control. This peptide was serial diluted to create a standard curve (*in duplo*). All samples were measured both undiluted and diluted 2× and 4× with 0.1 M PBS. Samples and GR peptide were incubated on the plate for 1 h at room temperature. After washing, all wells were incubated for 1 h with biotinylated monoclonal anti-GR antibody at a final concentration of 0.25 µg/ml in PBS-Tween 20/1% BSA. After washing again, samples were incubated for 20 min with Streptavidin-HRP (R&D Systems) diluted 1:200 in PBS-Tween 20/1% BSA. Following extensive washing, samples were incubated with substrate reaction mix (R&D Systems) for 15 min and stopped using 2N H_2_SO_4_. Read-out was carried out using a plate reader (Varioskan) at 450 nm and 570 nm.

### C9ORF72 strand-specific RT-PCR

RNA isolation was performed on mouse frozen kidney tissue and frozen frontal cortex of two C9FTD patients. Tissue was homogenized in 500 µl RIPA buffer (150 mM NaCl; 5 mM EDTA; 50 mM Tris-HCl, pH 8.0; 1% Nonidet-P40; 0.5% sodium deoxycholate; and 0.1% SDS, pH 7.6) containing complete protease inhibitor (Roche), 3 mM DTT (Invitrogen) and 40 units of RNAse OUT (Invitrogen). RNA was isolated using TRIzol reagent (Invitrogen), according to manufacturer's instructions. Reverse transcription was performed with 250 ng of RNA using a SuperScriptIII cDNA Synthesis kit (Invitrogen), according to manufacturer's instructions. RNA was treated with DNase before cDNA synthesis. The following C9ORF72 strand-specific primers were used to generate cDNA: LK-ASORF-R, 5′-CGACTGGAGCACGAGGACACTGACGAGTGGGTGAGTGAGGAG-3′ (after repeat) (Zu et al., 2013); or LK-ASORF-R2, 5′-CGACTGGAGCACGAGGACACTGAGTAGCAAGCTCTGGAACTCAGGAGTCG-3′ (before repeat) (Liu et al. 2017; Rizzu et al. 2016). For C9 FTD patient samples, PCR was performed using LK specific primer, 5′-CGACTGGAGCACGAGGACACTGA-3′ and reverse primer, 5′-AGTCGCTAGAGGCGAAAGC-3′. For mouse samples, PCR was performed using LK specific primer, 5′-CGACTGGAGCACGAGGACACTGA-3′ and reverse primer, 5′-CTCCTCACTCACCCACTCG-3′. The PCR program was as follows: 4 min at 94°C, followed by 35 cycles of 45 s at 94°C, 45 s at 58°C and 90 s at 72°C, followed by 6 min at 72°C.

## Supplementary Material

Supplementary information
