# Supplementary material for: Inducible expression of human C9ORF72 36× G4C2 hexanucleotide repeats is sufficient to cause RAN translation and rapid muscular atrophy in mice
Source: Dis Model Mech. 2021 Feb 16;14(2):dmm044842. doi: 10.1242/dmm.044842 (PMC7903916; doi:10.1242/dmm.044842)
Supplement: Supplementary information [file dmm-14-044842-s1.pdf]

```
Mouse   17 ----GGA--------ATTCGCCCTTGtttttcccaccctctctCTcccCa
               |||        .||     ||||||||||||||  |||||||||||
Human  151 TTTAGGAGGTGTGTGTTT-----TTGTTTTTCCCACC--CTCTCTCCCCA

        55 ctacttgctcCCtcacagtactcgctgagggtgaacaagaaaagacctga
           |||||||||  |||||||||||||||||||||||||||||||||||||||
       194 CTACTTGCT--CTCACAGTACTCGCTGAGGGTGAACAAGAAAAGACCTGA

       105 taaagattaaccagaagaaaacaaggagggaaacaaccgcagcctgtagc
           ||||||||||||||||||||||||||||||||||||||||||||||||||
       242 TAAAGATTAACCAGAAGAAAACAAGGAGGGAAACAACCGCAGCCTGTAGC

       155 aagctctggaactcaggagtcgcgcgctaggggccggggccggggccggg
           |||||||||||||||||||||||||||||
       292 AAGCTCTGGAACTCAGGAGTCGCGCGCTA---------------------

       205 gccggggccggggccggggccggggccggggccggggccggggccggggc

       321 --------------------------------------------------

       255 cggggccggggccggggccggggccggggccggggccggggccggggccg

       321 --------------------------------------------------

       305 gggccggggccggggccggggccggggccggggccggggccggggccggg

       321 --------------------------------------------------

       355 gccggggccggggccggggccggggccggggccggggccggggccggggc
                                      |||||||||||||||||||||||
       321 ---------------------------GGGGCCGGGGCCGGGGCCGGGGC

       405 gtggtcggggcgggcccgggggcgggcccggggcggggctgcggttgcgg
           ||||||||||||||||||||||||||||||||||||||||||||||||||
       344 GTGGTCGGGGCGGGCCCGGGGGCGGGCCCGGGGCGGGGCTGCGGTTGCGG

       455 tgcctgcgcccgcggcggcggaggcgcaggcggtggcgagtgggtgagtg
           ||||||||||||||||||||||||||||||||||||||||||||||||||
       394 TGCCTGCGCCCGCGGCGGCGGAGGCGCAGGCGGTGGCGAGTGGGTGAGTG

       505 aggaggc--------ggcggg--------------------cccggga--
           |||||||        ||||||                    .||||||
       444 AGGAGGCGGCATCCTGGCGGGTGGCTGTTTGGGGTTCGGCTGCCGGGAAG
```

Figure S1: Alignment of DNA sequence of mouse transgene (lower case) and human C9ORF72 sequence (upper case/ capital letters) surrounding the repeat expansion. NCBI Reference Sequence: NG_031977.1. Our mouse model contains 118 bp upstream and 115 bp downstream human flanking region around the G4C2 repeat expansion.

Disease Models & Mechanisms • Supplementary information

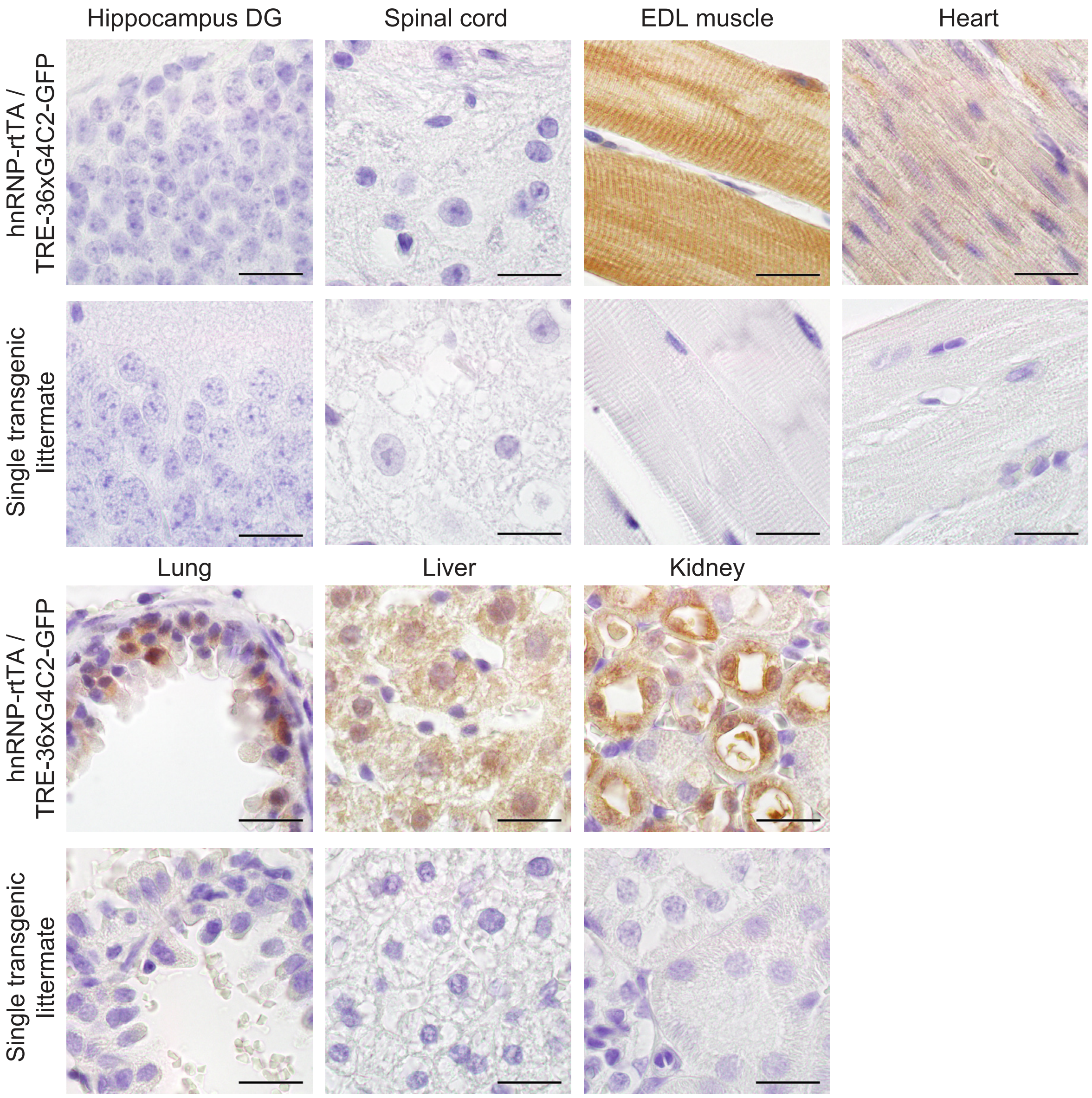

Figure S2: GFP expression in EDL muscle, heart, lung, liver and kidney of TRE-36xG4C2-GFP/hnRNP-rtTA double transgenic mice. No GFP staining was observed in the hippocampus dentate gyrus or in the spinal cord of DT mice. Single transgenic littermates, consist-ing of either TRE-only or rtTA-only, received the same dox treatment and are all negative for GFP staining. Scale bars are 20 µm. All stainings were performed on all mice in this study. ST 4 weeks dox n=15, DT 4 weeks dox n=16

Disease Models & Mechanisms • Supplementary information

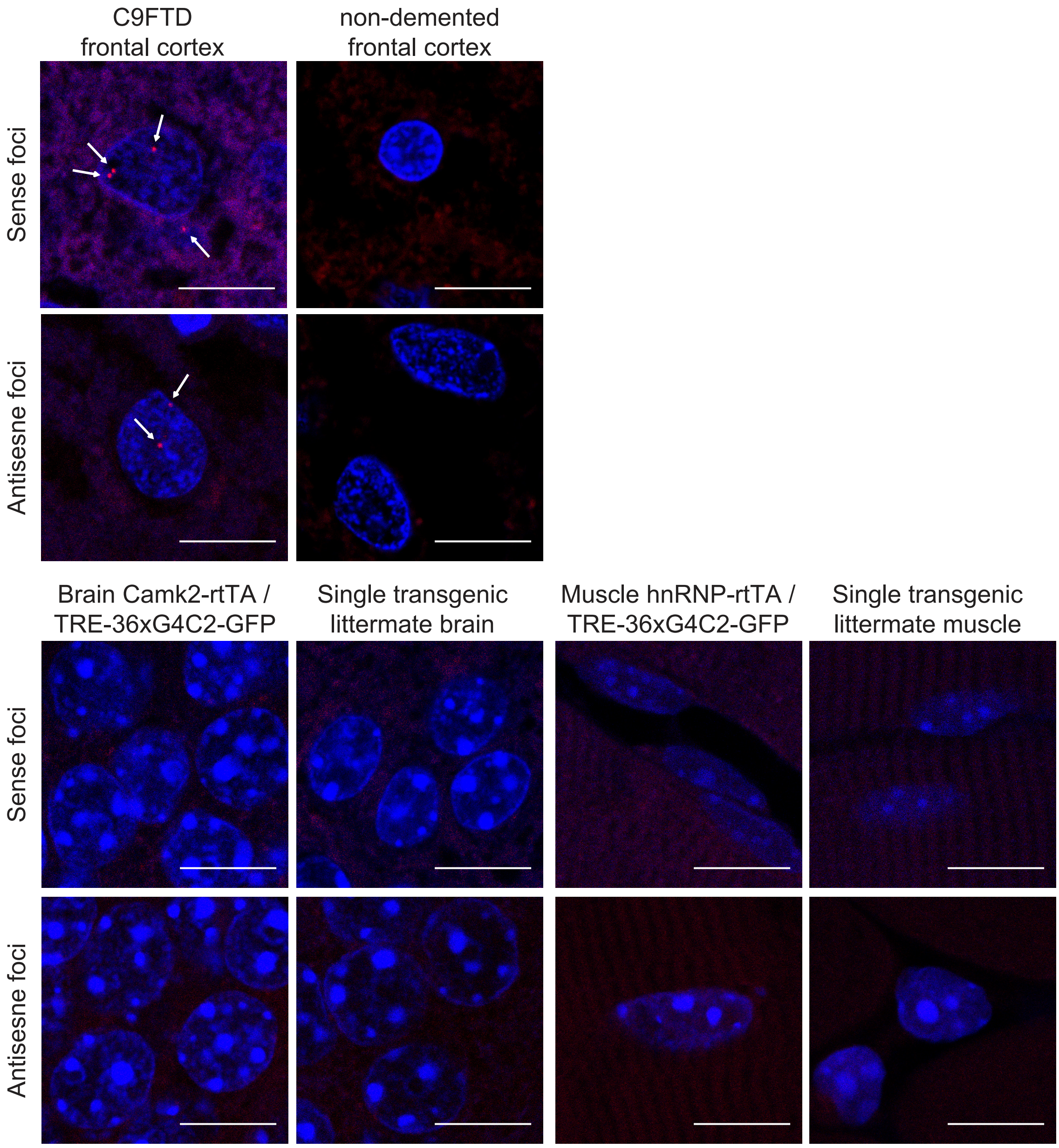

Figure S3: No sense nor antisense RNA foci were found in TRE-36xG4C2-GFP/Camk2-alpha-rtTA and TRE-36xG4C2-GFP/hnRNP-rtTA double transgenic mice and control single transgenic littermates. Single and double transgenic mice received the same dox treatment. Only frontal cortex samples of C9FTD cases present with some nuclear sense and antisense foci. Scale bars are 10 μm. The FISH was performed on all mice in this study. ST 4 weeks dox n=15, DT 4 weeks dox n=16

A rt-PCR for C9orf72 antisense transcipts on human prefrontal cortex samples

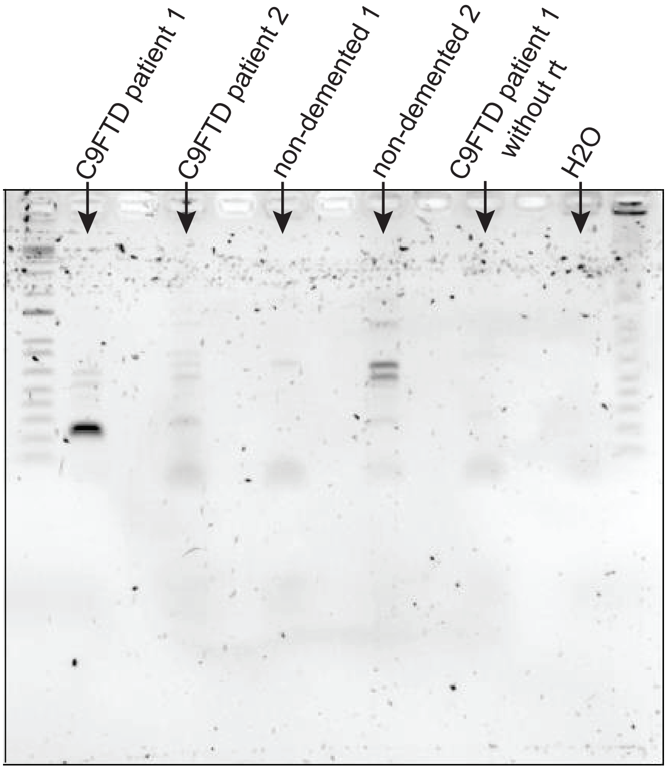

B rt-PCR for C9orf72 antisense transcipts on TRE-36xG4C2-GFP mouse kidney samples

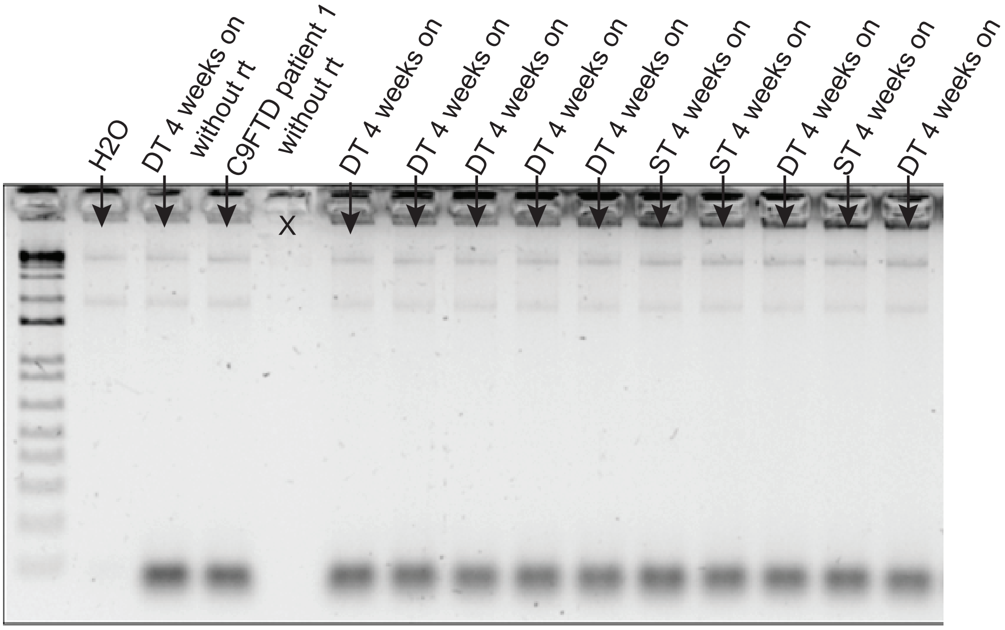

Figure S4: reverse transcriptase PCR for C9orf72 antisense tran-scripts. A) rt-PCR for C9orf72 antisense transcripts on frozen human prefrontal cortex samples with C9orf72 reverse specific primers. PCR product is only expected in C9ALS/FTD patients and is around 170bp without repeat, longer for the small repeat in C9FTD patient 1 and absent due to unknown reasons in C9FTD patient 2. Non-demented controls and samples run without reverse transcriptase show no bands (as expected) or some a-specific bands. B) rt-PCR for C9orf72 antisense transcripts on TRE-36xG4C2-GFP mouse kidney samples with C9orf72 reverse specific primers. PCR product is only expected in double transgenic (DT) mouse samples if any antisense transcription would be present, the PCR product would be around 360bp including the 36 repeat. Single transgenic (ST) and samples without reverse transcriptase (rt) are empty except for primer dimers (as expected). Lane number 4 is left empty because of broken well.

Disease Models & Mechanisms • Supplementary information

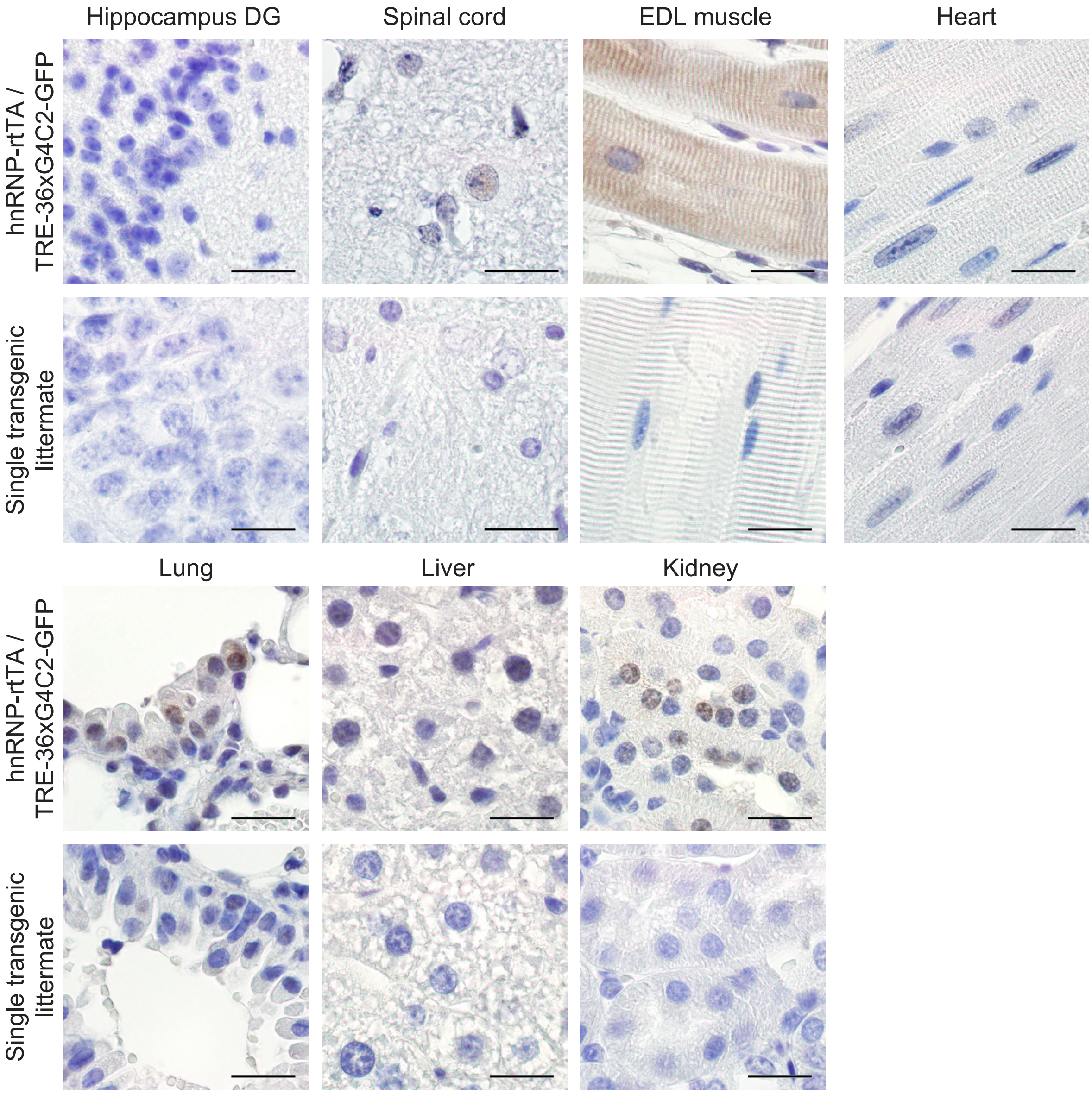

Figure S5: Poly-GA expression in EDL muscle, lung, liver and kidney of TRE-36xG4C2-GFP/hnRNP-rtTA double transgenic mice. No poly-GA staining was observed in the hippocampus dentate gyrus or in the spinal cord of DT mice. Single transgenic littermates, consisting of either TRE-only or rtTA-only, were treated similarly with dox and are all negative for poly-GA staining. Scale bars are 20 μm. The poly-GA was performed on all mice in this study. ST 4 weeks dox n=15, DT 4 weeks dox n=16

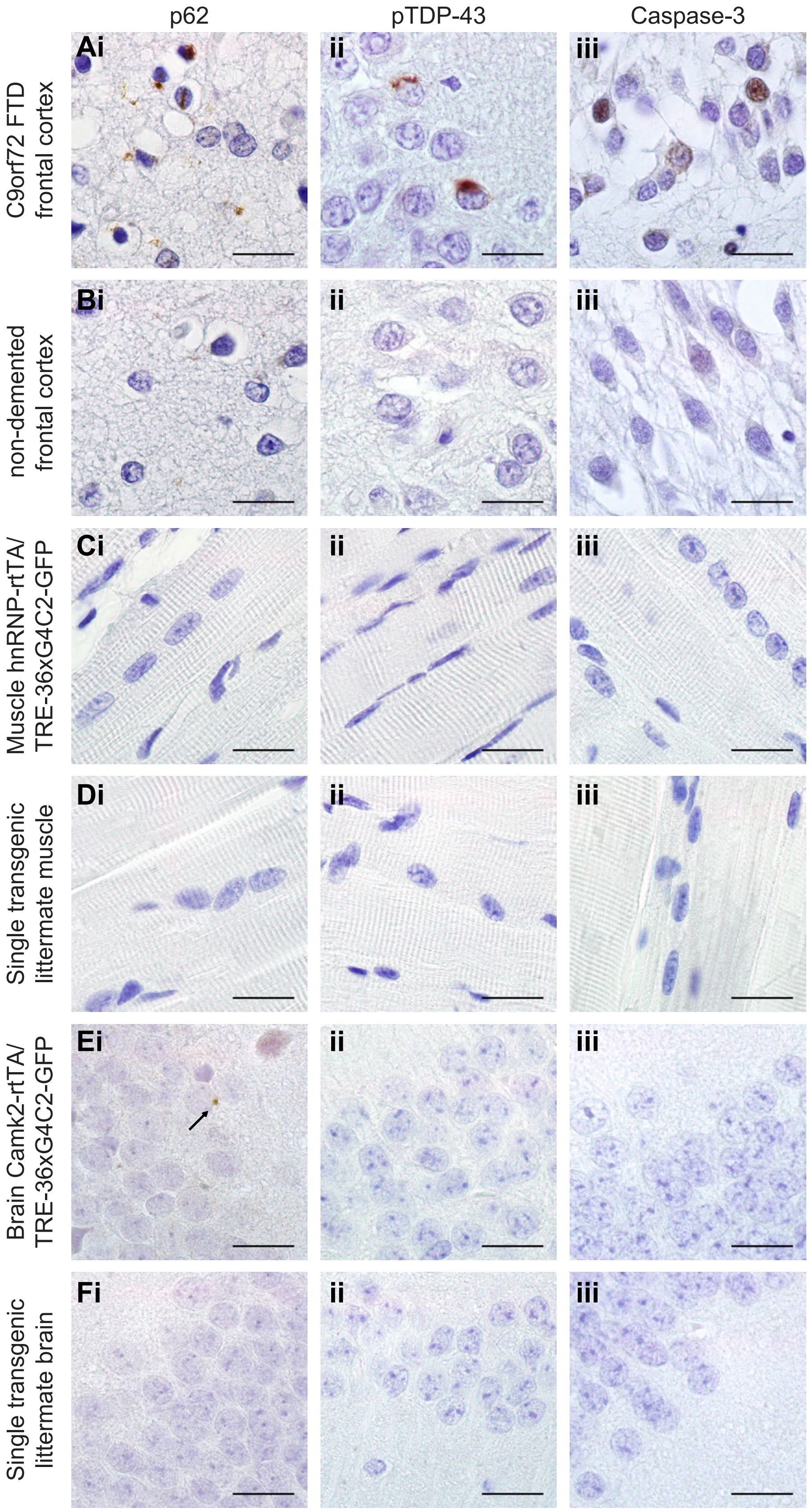

Figure S6: Expression of 36x G4C2 human repeats does not cause abundant p62, pTDP-43 and cleaved-caspase 3 pathology. Human prefrontal cortex of A) C9FTD patients or B) non-demented controls were used as positive and negative control for detection of pathology. C) TRE-36xG4C2-GFP/hnRNP-rtTA double transgenic mice do not present with any p62, pTDP-43 or cleaved-caspase-3 pathology in EDL muscle. E) TRE-36xG4C2-GFP/Camk2-alpha-rtTA double transgenic mice show some sparse perinuclear aggregates of p62 in the hippocampus dentate gyrus (arrow). D) and F) Single transgenic littermates, consisting of either TRE-only or rtTA-only, were treated similarly with dox and are negative for all pathology. All scale bars are 20 µm. All stainings were performed on all mice in this study. ST 4 weeks dox n=15, DT 4 weeks dox n=16.

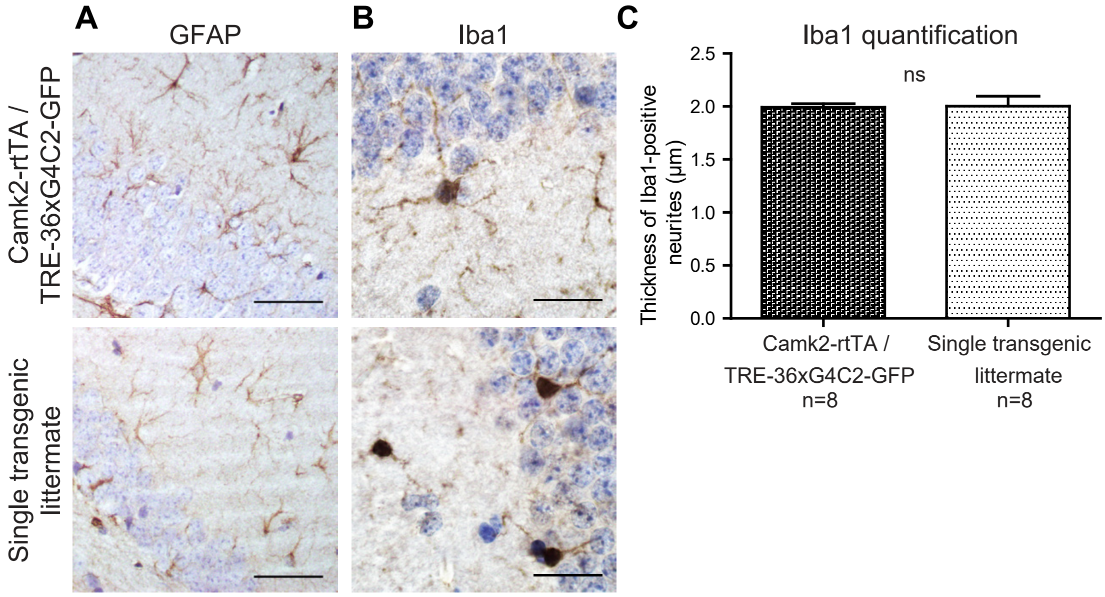

Figure S7: TRE-36xG4C2-GFP/Camk2-alpha-rtTA double transgenic mice do not show astrogliosis or microgliosis. A) Astrogliosis was assessed with GFAP labeling and B) microgliosis was tested with Iba1 staining. No differences in amount or morphology of GFAP-positive and Iba1-positive cells were seen in the hippocampus dentate gyrus of TRE-36xG4C2-GFP/Camk2-alpha-rtTA double transgenic mice and single transgen-ic control littermates. Single transgenic littermates, consisting of either TRE-only or rtTA-only, were treated similarly with dox. Scale bars are 20 µm. C) To quantify the thickness of Iba1-positive neurites, averages were taken of 10 pictures per mouse. N=8 TRE-36xG4C2-GFP/Camk2-alpha-rtTA double transgenic mice and n=8 single transgenic controls. T-test p=0.9099.

Disease Models & Mechanisms • Supplementary information

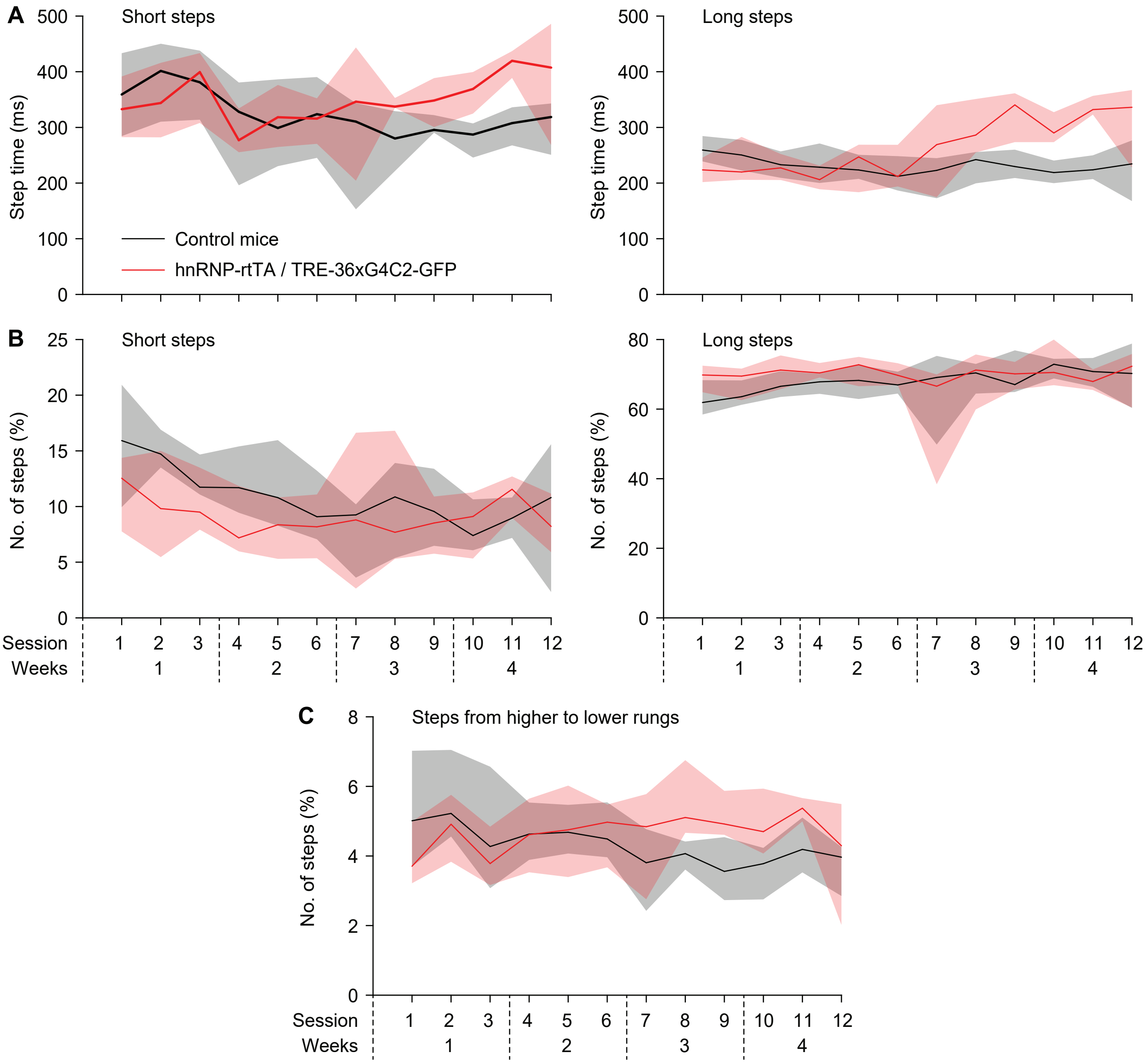

Figure S8: Erasmus ladder readouts. A) Step times of short steps (from one higher rung to the next; left) and long steps (skipping one higher rung, right). B) Fraction of short and long steps of all steps. C) Fraction of steps that were made from a higher rung to a lower rung. Lines indicate medians and shaded areas the interquartile ranges.

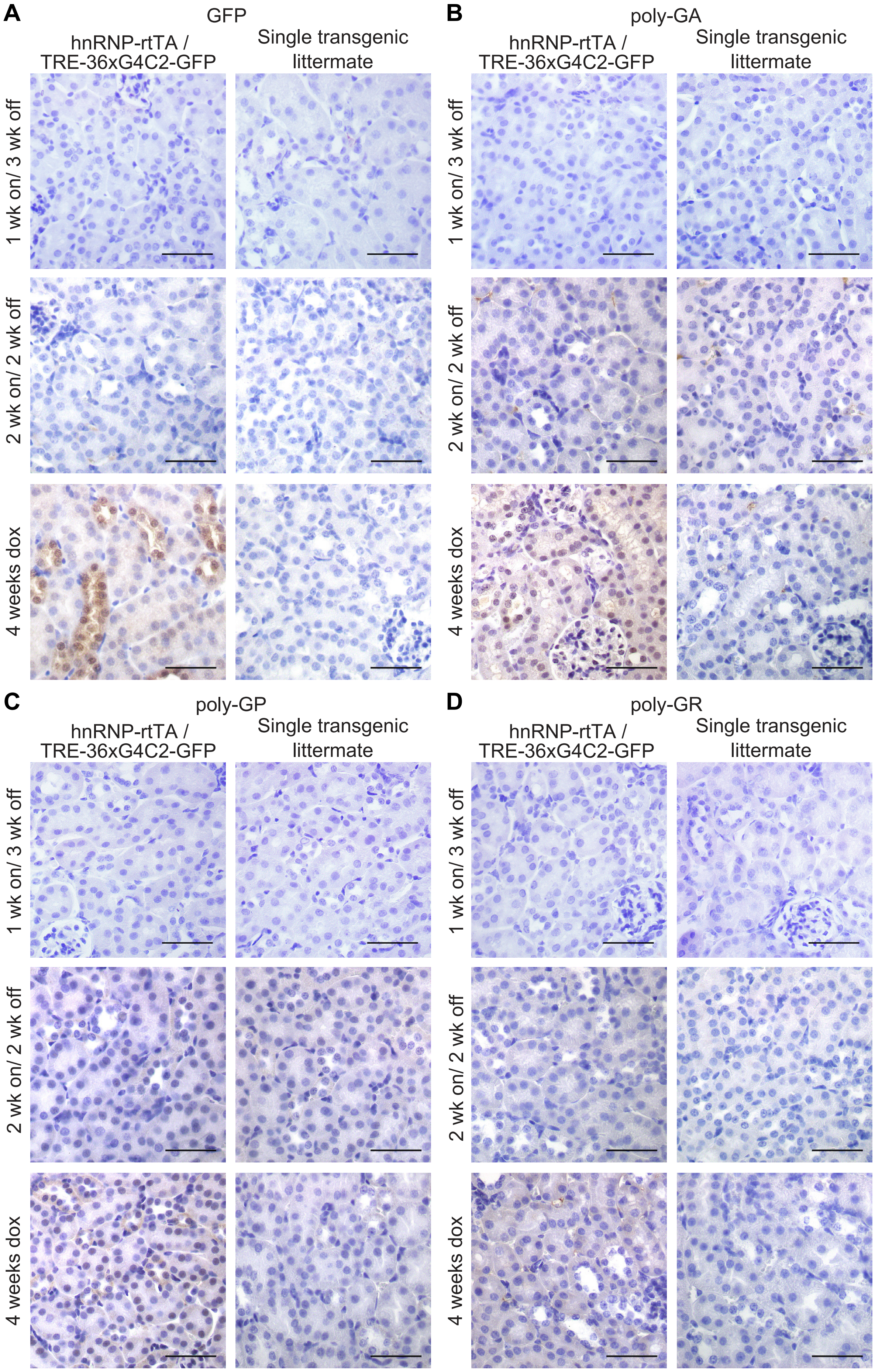

Figure S9: GFP and sense DPRs are cleared from the kidney after 2 weeks of dox withdrawal. A) GFP staining on kidney of TRE-36xG4C2-GFP/hnRNP-rtTA double transgenic mice shows clearance of GFP staining when mice received 2 weeks of dox water followed by 2 weeks of normal drinking water compared to DT littermates that received 4 weeks of dox. B) Poly-GA staining of kidney shows clearance of poly-GA after two weeks of dox withdrawal. C) Poly-GP staining and D) Poly-GR staining are also cleared from kidneys after 2 weeks of dox withdrawal. Single transgenic littermates received 2 or 4 weeks of dox and are all negative for GFP and DPRs. All scale bars are 50 µm. All stainings were performed on all mice in this study. Num-bers per group are: ST 1 week dox n=7, DT 1 week dox n=8, ST 1 week on/3 weeks off n=4, DT 1 week on/3 weeks off n=7, ST 2 weeks dox n=6, DT 2 weeks dox n=8, ST 2 weeks on/2 weeks off n=6, DT 2 weeks on/2 weeks off n=5, ST 4 weeks dox n=15, DT 4 weeks dox n=16

Table S1. Antibodies

Ab name	Host	Company	Cat.nr	Dilution
GA	mouse	Millipore, clone 5E9	MABN889	1:500
GP	rabbit	Bio Connect Life Sciences	24494-1-AP	1:250
GR	mouse	LifeTein Services	n.a. (costum-made)	1:4000
PR	mouse	LifeTein Services	n.a. (costum-made)	1:500
PA	mouse	Gift from Petrucelli	n.a.	1:2500
pTDP-43	mouse	Cosmo bio	CAC-TIP-PTD-M01	1:1000
p62	mouse	BD Biosciences	610833	1:100
Neurofilament	chicken	2BScientific Ltd.	CPCA-NF-H-25ul	1:500
GFAP	Rabbit	Sigma	G-9269	1:100
Iba1	rabbit	Wako	019-19741	1:200
ChAT	goat	Chemicon	AB144P	1:500
poly-HRP anti Ms/Rb IgG	goat	Immunologic	DPV055HRP	undiluted
anti-mouse HRP	goat	DAKO	P0260	1:100
anti-rabbit HRP	goat	DAKO	P0217	1:100
anti-mouse Cy2	goat	Jackson	715-255-150	1:100
anti-rabbit Cy3	goat	Jackson	711-165-152	1:100
anti-chicken 488	goat	Jackson	303-545-006	1:100
anti-goat HRP	rabbit	DAKO	P0449	1:100

Disease Models & Mechanisms • Supplementary information
